# Altered automatic gaze processing in older adults

**DOI:** 10.3389/fnagi.2025.1592763

**Published:** 2025-07-08

**Authors:** Roger Koenig-Robert, Boris Barrientos, Phoebe E. Bailey, Kiley Seymour

**Affiliations:** ^1^Faculty of Health, University of Technology Sydney, Sydney, NSW, Australia; ^2^School of Psychology, The University of New South Wales, Kensington, NSW, Australia; ^3^The MARCS Institute, Western Sydney University, Westmead, NSW, Australia

**Keywords:** gaze perception, continuous flash suppression, cognitive deficit, older adults, social abilities

## Abstract

From understanding others’ mental states to interpreting social cues, aging impairs social abilities. These impairments might not seem surprising given they rely on other cognitive functions such as memory, attention and decision-making, which are known to decline with age. It is, however, unclear to what degree impairments of more basic perceptual abilities, such as eye-gaze detection, contribute to or even precede the decline in social skills. Previous studies have obtained mixed results when investigating whether aging impairs fundamental perceptual processing of social information. Our study expands on previous findings by showing that aging impairs the ability to rapidly detect and discriminate gaze direction. Using breaking Continuous Flash Suppression (b-CFS), we tested whether preconscious automatic processing of direct eye contact was prioritized over the processing of averted gaze direction, as previously established in younger adults. Our results show that, on average, older adults (65–89 years old, *n* = 19) lack this direct gaze advantage and do not exhibit significant differences in detecting direct vs. averted gaze direction. These results provide important insights into age-related deficits in social cognition, suggesting social processing deficits may manifest at the earliest automatic stages of perceptual processing. Future work examining the relationship between alterations in gaze processing and decline in higher-level cognitive functions could inform the development of early detection tools and clinical interventions.

## Introduction

Age-related changes significantly affect aspects of social abilities (Fernandes et al., 2021). Older adults show difficulties in interpreting and predicting others’ behavior and intentions (Sullivan and Ruffman, 2004), perceiving and processing social cues (Slessor et al., 2008), as well as recognizing negative emotions such as anger, fear, and sadness (Ruffman et al., 2008). Age-related deficits in social cognition can lead to significant challenges in maintaining social connections, contributing to a decrease in overall life satisfaction, and exacerbating mental health issues such as depression and anxiety (Nicholson, 2012; Wang et al., 2018).

Social abilities depend on several higher-level cognitive functions, such as attention, memory, language, and decision-making, which are well-documented to decline with age (Baghel et al., 2019; Li et al., 2001; Mather, 2010). It is, therefore, not surprising that older adults struggle with social abilities as their cognition declines. However, beyond the decline of high-level cognitive abilities, there is limited research on whether other factors contribute to or even precede the decline in social abilities in older adults. The effect of aging on more basic perceptual processing of social cues has been underexplored. One of the most basic perceptual mechanisms of social processing is detecting the gaze of others (Emery, 2000). The detection of eye gaze direction (i.e., detecting whether someone is looking at you or away) is thought to involve a three-step process of identifying eyes or eye-like stimuli in the environment, processing gaze direction, and interpreting eye-gaze as seeing (Baron-Cohen, 1995). Information about the direction of someone’s gaze offers an observer a way to assess what other people are focusing on (such as the location of an object) and is an important social cue that draws attention to important elements in the environment (Driver et al., 1999), a phenomenon commonly known as joint attention (Mundy, 2018).

A previous study has shown that older adults exhibit deficits in joint attention compared to young adults (Slessor et al., 2008). These results suggest that basic functions associated with social perception may also decline with age alongside impairments in higher-level cognition. An outstanding question from this research is whether deficits in joint attention can be explained by alterations at even earlier stages of perceptual processes that involve the initial preconscious and more automatic stages of detecting eyes in the environment to enable gaze direction discrimination. Indeed, a recent study investigated whether subliminal (unconscious) stimuli could trigger joint attention in older adults (Bailey et al., 2014). The study revealed that older adults showed significant gaze-cueing effects (i.e., enhanced attentional orienting towards the direction of gaze) in both the supraliminal and subliminal conditions, but the effects were significantly weaker compared to younger participants (Bailey et al., 2014). These findings may indicate that early stages of gaze processing are impacted by age. However, whether preconscious stages of gaze detection and discrimination are affected warrants further investigation.

**Figure 1:**
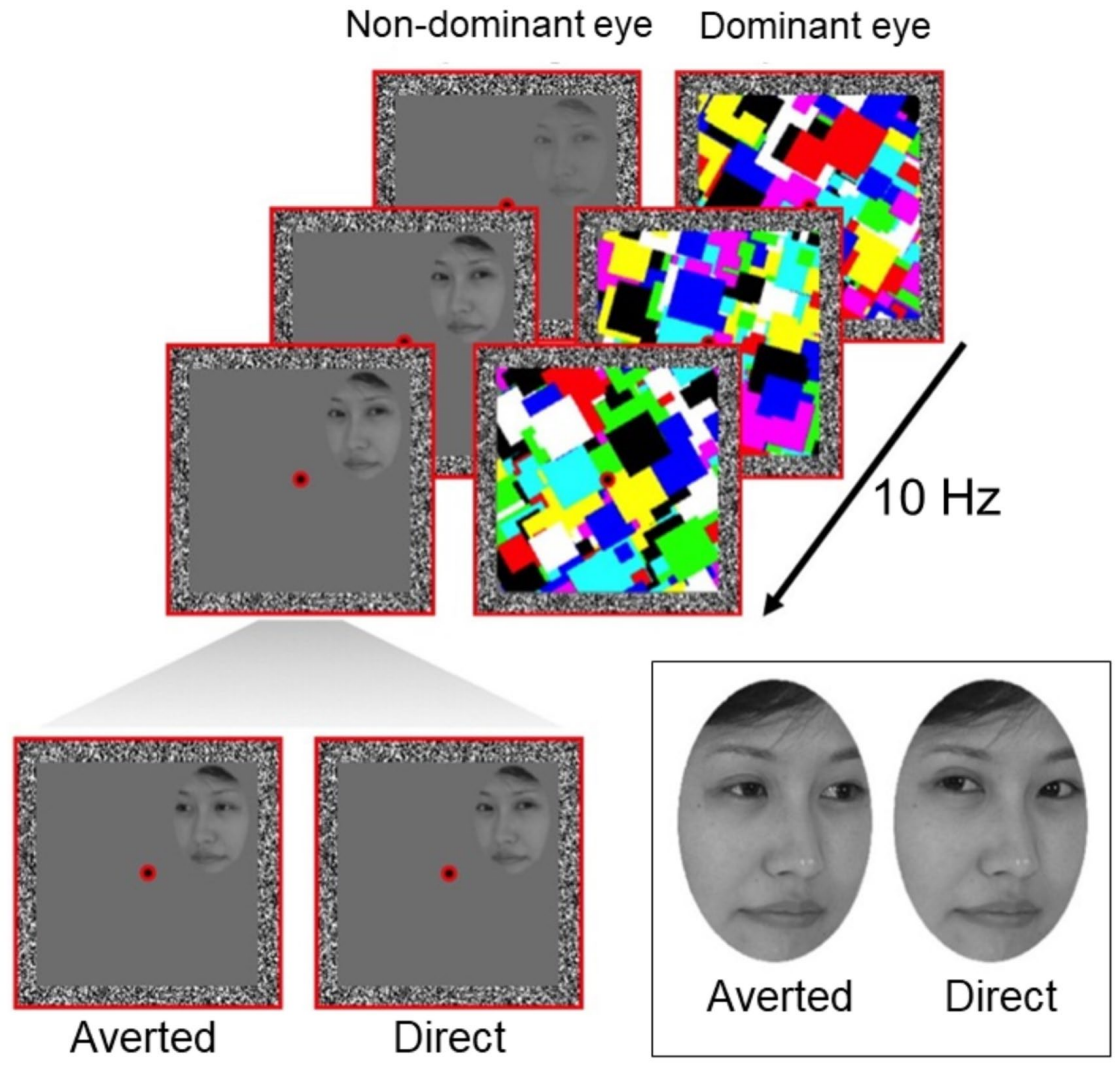
Schematic of a b-CFS trial. A dynamic mask is presented to the dominant eye, temporarily suppressing the face stimulus presented to the non-dominant eye from reaching conscious awareness. Participants are required to indicate on which side of the central fixation point the face breaks suppression and becomes visible. Faces are presented with either direct gaze or averted gaze. Face stimuli used originates from Senju and Hasegawa (2005).

In the current study, we investigated whether preconscious gaze processing is affected by age. Using a technique known as breaking continuous flash suppression or b-CFS (Stein et al., 2011a; Tsuchiya and Koch, 2005), we temporarily suppressed photographs of faces from visual awareness (Figure 1). In this paradigm, the time taken for the face to break through the suppressive mask and become visible to the participant is typically treated as an index of its salience or its preconscious perceptual prioritization (Kaliuzhna et al., 2019; Seymour et al., 2016; Seymour et al., 2024; Stein et al., 2011b). Participants had to report the moment when they detected the face and on which side of the screen (left or right) they detected it, which allowed us to control for participants performing the task correctly. Previous experiments using b-CFS have consistently shown shorter reaction times to detect suppressed faces with direct gaze compared to averted gaze, suggesting that the visual system prioritizes the detection of faces with direct gaze (Seymour et al., 2016; Stein et al., 2011b; Yokoyama et al., 2013). We examined whether older adults show this same processing advantage for faces with direct eye gaze. We hypothesized that if age impairs automatic preconscious processing of gaze, we should observe a reduced processing advantage for direct eye gaze in older participants.

## Materials and methods

### Participants

A total of 32 older adults (65 + years old; 17F) and 29 undergraduate students (18–35 years old; 17F) from Western Sydney University participated in the study. The undergraduate participants were recruited through Western Sydney University’s SONA system, and older adults were recruited from the community. Undergraduate students received course credit for their time. Older adults received $30. All participants signed an informed consent form, which was approved by the human research ethics committee at Western Sydney University (reference number H12571). Participants with localization accuracies (correctly reporting the side, left or right, where the face was presented on the screen) under 70% were removed (thirteen older and nine young participants), leaving 19 older and 20 young participants. This sample size was comparable to group studies using related paradigms (Seymour et al., 2016; Seymour et al., 2024), resulting in a post-hoc power (1-*β*) of 0.86 (*α* = 0.05, one-sided) for the gaze direction main effect.

### Apparatus and stimuli

We closely followed a b-CFS protocol previously used to measure automatic and unconscious processing of eye gaze direction in human participants (Stein et al., 2011b), as shown in Figure 1. Stimuli were viewed on a CRT computer monitor through a mirror stereoscope (resolution: 1024 × 768, 60 Hz). Two adjacent red square frames (10.6° x 10.6°) were displayed such that only one frame was visible to each eye. We confirmed this with participants by asking them to report what they saw when they viewed the stimulus monocularly before testing. In the center of each frame, a red fixation dot was presented. Fusion contours (width 0.8°) consisting of random noise pixels were also presented at the border of each frame to support binocular fusion of the two eyes’ images.

During the task, we presented face stimuli previously used in b-CFS experiments (Senju and Hasegawa, 2005). These faces were monochrome digital photographs of four adult females with neutral facial expressions. Eye gaze was directed straight ahead (direct gaze) or away (averted gaze). All faces were equally adjusted in contrast and brightness and displayed in an oval (3.3° x 4.6°) to obscure hairlines. Edges of this aperture were blurred to assist in suppressing the face stimuli during CFS masking.

### Procedure

Participants maintained fixation on the central fixation point throughout the task. Each trial began with a 1 s presentation of the red frames, fusion contours, and fixation dots on a uniform black background. Following this, a suppressive multi-colored Mondrian mask (updating at a frequency of 10 Hz) was introduced to the dominant eye (confirmed using the near convergence test, Rice et al., 2008) and gradually ramped down linearly from 100% stimulus contrast to 0% stimulus contrast from 7 to 12 s, which was the total trial length. In the non-dominant eye, a face stimulus was gradually introduced by ramping up its contrast linearly from 0 to 100% over a 1 s period. Participants were required to indicate whether the face stimulus was presented to the left or right of the central fixation point via arrow keys on the keyboard, note that each side had the same probability of showing direct and averted gaze. No specific response about the gaze direction was required. This is because b-CFS probes early stages of perception and does not require participants to report the features of the stimulus; the use of this method specifically eliminates potential influences of cognitive bias on the participant’s report (Caruana et al., 2019a,b; Caruana and Seymour, 2021; Seymour et al., 2016). In half of the trials, face stimuli were presented to the left of fixation (horizontal center-to-center distance 2.7°). In the other half of the trials, faces were presented to the right. The order of the trials was pseudorandomized. Participants were instructed to respond as soon as they located any part of the face and to indicate whether it was located left or right of fixation. The time taken to make a response was used to indicate how long the stimulus took to break through the suppressive mask and reach conscious awareness (Tsuchiya and Koch, 2005; Yang et al., 2007). Shorter suppression times were taken to indicate faster preconscious processing and prioritization of that stimulus by the visual system. Location accuracy was measured on each trial, which allowed us to ascertain whether participants performed the task correctly. Only participants with accuracies above 70% and only correct trials from these participants were considered for analysis.

Participants completed 144 trials (72 direct gaze and 72 averted gaze), separated evenly into four blocks. Suppression times for each trial were recorded. Mean suppression times were calculated for direct and averted gaze stimuli (only on correct trials). Throughout the task, participants were seated 57 cm from the screen with their heads stabilized with a chin rest.

We removed trials with suppression break times longer than 10s (3 s after the Mondrian pattern started to ramp down from full contrast, corresponding to 40% contrast for the Mondrian mask and deemed to be too low to be effectively suppressing the face stimulus) and shorter than 100 ms, which were deemed too long and quick, respectively, to indicate meaningful responses (Gayet et al., 2016; Slessor et al., 2008, 2010; Stein et al., 2011a). The percentage of such responses was low in our sample 8.2% of trials, corresponding to 11.8 (±5.1 SD) out of 144 trials per participant on average.

### Autism-spectrum and empathy questionnaires

After the b-CFS experiment, participants completed the Autism-spectrum Quotient (AQ) questionnaire (Baron-Cohen et al., 2001b) and the Empathy Quotient (EQ) questionnaire (Baron-Cohen and Wheelwright, 2004). These measures allowed us to probe whether any differences observed in the size of the direct gaze advantage effect were related to self-reported difficulties in social skills and understanding others’ mental states. Extreme scores for AQ and EQ questionnaires have been linked with social functioning deficits in clinical and non-clinical populations (Baron-Cohen et al., 2001a; Bird and Viding, 2014; Losh and Capps, 2006).

### Statistical analyses

Statistical analyses were conducted using the Statistics Toolbox from MATLAB version 2024b Natick, Massachusetts: The MathWorks Inc.; 2024 and the bayesFactor Toolbox version 3 (Krekelberg, 2024) for MATLAB. The effects of our two gaze directions on mean suppression times across two groups (young and older adults) were examined using a mixed-design ANOVA with gaze direction (direct vs. averted) as a within-subject factor and group (young vs. older) as a between-subject factor. In line with previous literature, we used suppression-breaking times as an index of stimulus potency in reaching conscious awareness (Caruana et al., 2019a; Gayet et al., 2014; Jiang et al., 2007; Stein et al., 2011a). We further calculated the direct gaze advantage score as the difference between the breaking suppression times for the averted gaze condition and the direct gaze condition (Caruana et al., 2019b; Jackson and Seymour, 2022; Seymour et al., 2016). The calculation of the direct gaze advantage score has been used in previous literature to address inter-subject variability in RT, as well as possible group differences (Caruana et al., 2019b; Jackson and Seymour, 2022). Post-hoc tests were used to make pairwise statistical comparisons. We employed *t*-test, from the Statistics Toolbox from MATLAB, and Bayes factor as BF10, using the bayesFactor Toolbox version 3 for MATLAB. Bayes factors were derived from T-values as detailed in Rouder et al. (2012) and Morey and Wagenmakers (2014).

## Results

### Direct gaze advantage in young and older participants

A mixed-design ANOVA with gaze direction (direct vs. averted) as within-subject and group (young vs. older) as between-subject factor revealed a significant effect of age on overall suppression times (F(1,37) = 6.34, *p* = 0.016); older participants were generally slower to detect either stimulus category (3,837 ms ± 23, Mean ±SEM) compared to younger participants (3,075 ms ± 24, Mean ±SEM). We also found a significant main effect of gaze direction (F(1,37) = 7.79, *p* = 0.008), with the detection of direct gaze being prioritised over averted, thus replicating previous results of a direct gaze advantage with b-CFS (Kaliuzhna et al., 2019; Seymour et al., 2016, 2024; Stein et al., 2011b). However, no significant interaction between age and gaze direction was observed (F(1,37) = 0.393, *p* = 0.53).

**Figure 2:**
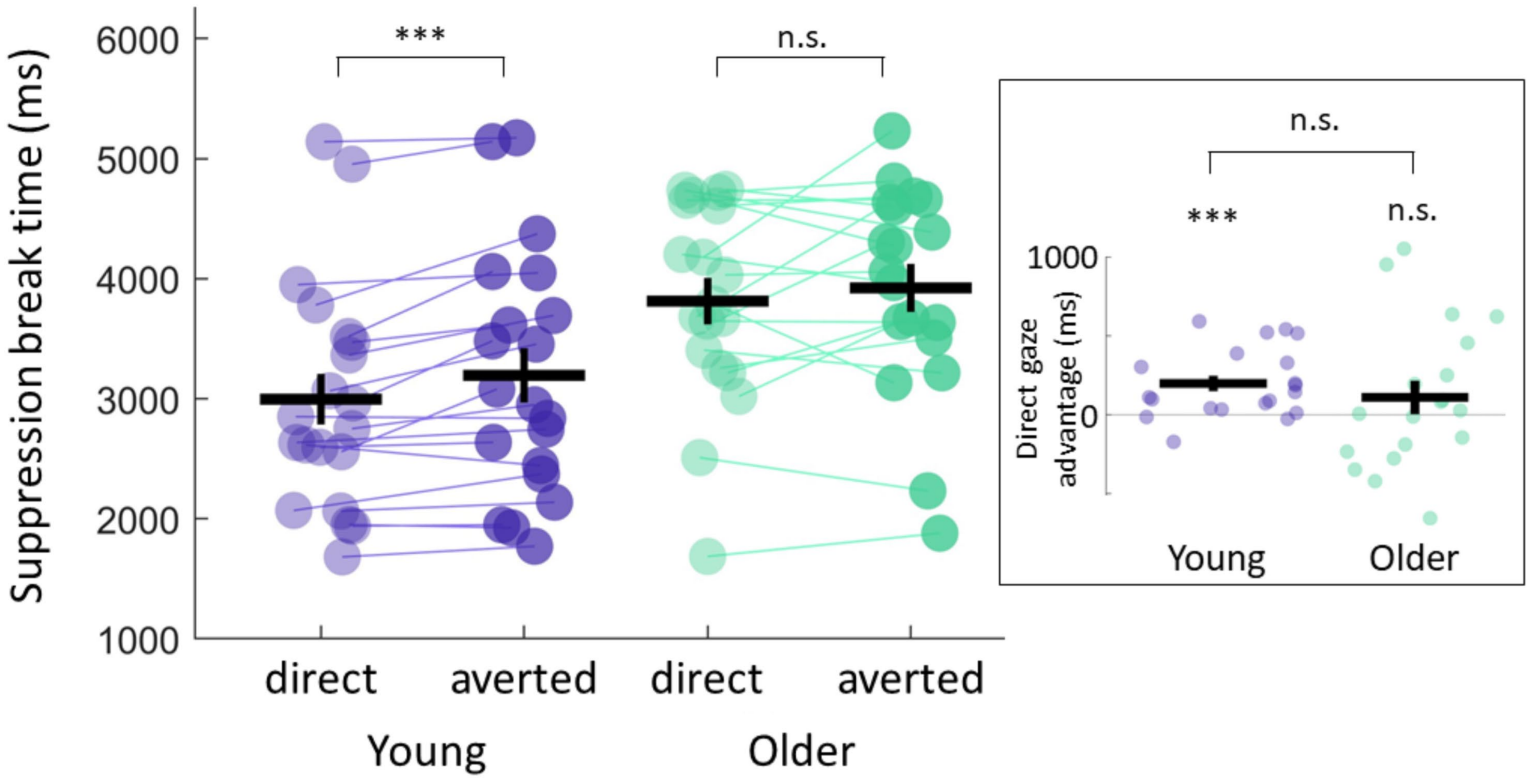
Direct gaze advantage. Young participants showed shorter suppression break times for direct gaze stimuli and a significant direct gaze advantage (averted-direct RT) of 197 ms ± 50, Mean ±SEM (t(19) = 3.97, *p* = 0.0008, CI = [93.3, 301.1], one-sample *t*-test against zero, bf10 = 43.59). Older participants only showed a trend for faster direct gaze detection with a direct gaze advantage of 124 ms ± 106, which was not significantly different from zero (t(18) = 1.17, *p* = 0.25, CI = [−98.3, 347.9], one sample t-test against zero, Bayes factor bf10 = 0.43). Data from individual participants is represented with lines linking participants’ data between the two conditions. Mean and standard error of the mean across participants in each group for each condition are also represented. The inset shows the size of the direct gaze advantage for each participant within each group. Positive values represent shorter suppression break times for direct gaze stimuli relative to averted gaze stimuli.

Although these data suggest no group difference in preconscious prioritization of direct gaze, we did find that only 57% of older participants showed the direct gaze advantage in comparison to 85% of control participants. By calculating the size of the direct gaze advantage for each participant, we effectively normalised our data to account for overall mean differences in reaction times observed between groups. We found that on average, young participants were significantly faster at detecting direct vs. averted eye gaze (2,977 ms ± 216 vs. 3,174 ms ± 232, Mean ±SEM, t(19) = 3.97, *p* = 0.0008, CI = [93.3, 301.1], paired t-test, Bayes factor bf10 = 43.59) with a direct gaze advantage (averted-direct RT) of 197 ms ± 50, Mean ±SEM (Figure 2). Our older participants, on the other hand, only showed a trend of shorter suppression times for direct gaze stimuli (3,775 ms ± 207 vs. 3,900 ms ± 213, Mean ±SEM); suppression times across conditions were not significantly different (t(18) = 1.17, *p* = 0.25, CI = [−98.3, 347.9], paired-t-test, Bayes factor bf10 = 0.43). Importantly, the size of the direct gaze advantage (averted-direct RT, see Methods for details) was small 124 ms ± 106 (Mean ±SEM) and not significantly different from zero (*p* = 0.25). Although we failed to show that the differences in the size of the direct gaze advantage were significantly different across groups (*p* = 0.53, two-sample t-test, bf10 = 0.36), our results do suggest that deficits in preconscious processing can be measured more frequently in older participants, but that there is a high degree of variability within this group.

### Altered gaze processing and its relationship to other measures of high-order social cognition

We subsequently sought to test whether the size of the direct gaze advantage had any relationship with self-reported abilities in social skills and understanding others’ mental states. We examined relationships with the Autism-spectrum Quotient (AQ) questionnaire (Baron-Cohen et al., 2001b) and the Empathy Quotient (EQ) questionnaire (Baron-Cohen and Wheelwright, 2004). Extreme scores for AQ and EQ questionnaires have previously been linked with social functioning deficits in clinical and non-clinical populations (Baron-Cohen et al., 2001a; Bird and Viding, 2014; Losh and Capps, 2006). Our results revealed no significant correlation between the size of the direct gaze advantage (averted-direct) and AQ (R^2^ = 0.007, F(37) = 0.26, *p* = 0.61) nor between the direct gaze advantage and EQ (R^2^ = 0.03, F(37) = 1.15, *p* = 0.29).

## Discussion

This study aimed to examine whether preconscious and automatic stages of eye gaze processing are affected by age. Previous research has obtained mixed results in measuring deficits in the early stages of gaze processing in older participants, with one study reporting deficits in joint attention in older participants (Slessor et al., 2008). Another study indicated that automatic and subliminal processing of eye-gaze is preserved, albeit weaker (Bailey et al., 2014). Our data revealed that, on average, older participants failed to show a direct gaze advantage, consistent with reported deficits in the early stages of gaze processing (Slessor et al., 2008). However, a high level of variability was also observed in this group, with many participants (57%) showing preconscious prioritization of direct gaze processing. This may suggest that age can affect early automatic stages of eye gaze perception in a high proportion of individuals but that, in some individuals, this capacity is preserved. Understanding the risk profile and protective factors that contribute to this variability would be an important avenue for future work.

It is important to highlight that recent studies have provided alternative explanations that do not rely on unconscious mechanisms for the advantage of certain face attributes over others during b-CFS (Lanfranco et al., 2022, 2023a, 2023b; Stein and Peelen, 2021). These studies indicate that some of the effects commonly attributed to unconscious processing under b-CFS could be, at least in part, explained by differences in decision criteria, which could obfuscate the interpretation of b-CFS in terms of purely unconscious perceptual grounds. Additional measures involving detection-discrimination dissociation paradigms have been advanced as a promising way to establish the contribution of unconscious perception and decision criteria (Lanfranco et al., 2022; Stein and Peelen, 2021). This, in addition to recent criticisms of CFS (Lanfranco et al., 2023b; Pournaghdali and Schwartz, 2020), justify caution when interpreting the results from b-CFS. However, research has shown that these post-conscious accounts still rely on very early post-conscious processing (Hedger et al., 2016; Moors et al., 2019; Stein and Peelen, 2021). All in all, this does not detract from our conclusion that automatic gaze processing is impaired in older adults, albeit this deficit might not be purely unconscious. Future work should extend the results presented here by using detection-discrimination dissociation paradigms to quantify the influence of post-conscious mechanisms (if any) on these effects.

Our results also show that variability in gaze processing at its earliest perceptual stages had no relationship with how participants evaluated their higher-order social skills and social cognitive abilities as measured with the Autism-spectrum Quotient (AQ) questionnaire and the Empathy Quotient (EQ) questionnaire (Baron-Cohen et al., 2001b; Baron-Cohen and Wheelwright, 2004). While high scores on these measures have previously been associated with altered gaze processing in clinical populations (Akechi et al., 2014; Black et al., 2020; Cowan et al., 2014; Pantelis and Kennedy, 2017), subjective measures of social cognition are inherently confounded and may not have been sensitive enough here. Indeed previous reports of social cognitive deficits in older participants have used more objective tasks, showing decreased accuracy in judging emotional expressions from images of faces (Murphy and Isaacowitz, 2010; Ruffman et al., 2008; Sullivan and Ruffman, 2004), difficulties in theory of mind assessed via performance on the reading the mind in the eyes task (Henry et al., 2013; Moran, 2013; Rakoczy et al., 2012), and reduced general perspective-taking abilities as assessed by false-belief, director and story-based tasks (Henry et al., 2013; Moran, 2013). Thus, our results may suggest that alterations in automatic gaze processing, as measured in a large proportion of our older participants using b-CFS, may arise before other social cognitive deficits manifest. However, future studies that examine relationships between the direct gaze advantage and performance on more objective social cognitive tasks will better establish if a more general perturbation in early perceptual processing precedes deficits in higher levels of social cognition. Such a finding could present new ways to detect and treat impairments in an early stage.

Regarding the mechanisms associated with these deficits, previous research has suggested that lower-level automatic processes compensate for the loss of higher-level cognitive processes that begin to diminish with age (Kalokerinos et al., 2015). This compensatory account of aging posits that, as people age, controlled (higher-order) processes that inhibit automatic ones become less effective, making automatic processes stronger. Our study found no evidence for this notion, as we would expect to see a heightened direct gaze advantage in older participants in our study. Instead, our data showed the opposite effect, which suggests deficits in automatic gaze processing are evident with aging.

The automatic preconscious processing of eye contact and the direct eye gaze advantage is supported by subcortical pathways facilitating the rapid detection and orienting to direct eye contact (Johnson, 2005; Pessoa and Adolphs, 2010; Senju and Johnson, 2009; Tamietto et al., 2012). This subcortical gaze-detection pathway involves the superior colliculus as well as the pulvinar and the amygdala, providing a fast means to detect and respond to important social cues. Our data suggest that many older participants might exhibit an impairment in these subcortical pathways. Subcortical anomalies in older age have been linked with cognitive impairment and dementia (Wang et al., 2024; Zhu et al., 2021) and most notably in Parkinson’s disease (Esposito et al., 2021; Surkont et al., 2021). Interestingly, abnormalities in subcortical structures can be detected in healthy-aged people before cognitive deficits can be detected (Paul et al., 2005; Pugh and Lipsitz, 2002; Roob et al., 1999; Walhovd et al., 2005). Thus, our results are suggestive of potential loss of function in subcortical structures due to age. Future research will determine the exact brain regions linked to the perceptual effects measured in our study and whether these can serve as early indicators of future impairments. Functional MRI would be uniquely suited to investigate which brain areas’ activity is correlated with the effects observed in our study, as has been done in other studies focusing on different cognitive decline related to aging (Anguera et al., 2011; Kennedy et al., 2015; Smith et al., 2001). Moreover, understanding the protective factors that contribute to the preservation of the direct gaze advantage in some older individuals may allow for new treatment interventions.

In conclusion, this study was the first to use a b-CFS task to examine automatic eye gaze processing in older adults. We found that the direct gaze advantage (i.e., faster awareness of direct gaze compared to averted gaze) was impaired in a high proportion of older adult participants, with the group, on average, showing an absence of the effect. Our results suggest that deficits in the automatic processing of eye gaze may contribute to or precede losses in more complex social abilities. However, further research relating our results to objective measures of social cognition is needed. As automatic gaze perception can be measured easily with tasks like b-CFS, and impairments may be detected before other social cognitive deficits manifest, this method could be used as a tool to assess the risk of cognitive decline and inform early clinical intervention. Future work to establish the relationship between alterations in gaze processing and decline in higher-level cognitive functions is an important extension of this work.

## Data Availability

The raw data supporting the conclusions of this article will be made available by the authors, without undue reservation.
